# Social Contact Networks and Mixing among Students in K-12 Schools in Pittsburgh, PA

**DOI:** 10.1371/journal.pone.0151139

**Published:** 2016-03-15

**Authors:** Hasan Guclu, Jonathan Read, Charles J. Vukotich, David D. Galloway, Hongjiang Gao, Jeanette J. Rainey, Amra Uzicanin, Shanta M. Zimmer, Derek A. T. Cummings

**Affiliations:** 1 Department of Health Policy and Management, Graduate School of Public Health, University of Pittsburgh, Pittsburgh, Pennsylvania, United States of America; 2 Public Health Dynamics Laboratory, Graduate School of Public Health, University of Pittsburgh, Pittsburgh, Pennsylvania, United States of America; 3 Department of Statistics, Faculty of Science, Istanbul Medeniyet University, Istanbul, Turkey; 4 Department of Epidemiology and Population Health, The Farr Institute @HeRC, Institute of Infection and Global Health, University of Liverpool, Liverpool, L69 3GL, United Kingdom; 5 Lancaster Medical School, Lancaster University, Lancaster, LA1 4YG, United Kingdom; 6 School of Medicine, University of Pittsburgh, Pittsburgh, Pennsylvania, United States of America; 7 Division of Global Migration and Quarantine, US Centers of Disease Control and Prevention, Atlanta, Georgia, United States of America; 8 Bloomberg School of Public Health, Johns Hopkins University, Baltimore, Maryland, United States of America; Centre de Physique Théorique, FRANCE

## Abstract

Students attending schools play an important role in the transmission of influenza. In this study, we present a social network analysis of contacts among 1,828 students in eight different schools in urban and suburban areas in and near Pittsburgh, Pennsylvania, United States of America, including elementary, elementary-middle, middle, and high schools. We collected social contact information of students who wore wireless sensor devices that regularly recorded other devices if they are within a distance of 3 meters. We analyzed these networks to identify patterns of proximal student interactions in different classes and grades, to describe community structure within the schools, and to assess the impact of the physical environment of schools on proximal contacts. In the elementary and middle schools, we observed a high number of intra-grade and intra-classroom contacts and a relatively low number of inter-grade contacts. However, in high schools, contact networks were well connected and mixed across grades. High modularity of lower grades suggests that assumptions of homogeneous mixing in epidemic models may be inappropriate; whereas lower modularity in high schools suggests that homogenous mixing assumptions may be more acceptable in these settings. The results suggest that interventions targeting subsets of classrooms may work better in elementary schools than high schools. Our work presents quantitative measures of age-specific, school-based contacts that can be used as the basis for constructing models of the transmission of infections in schools.

## Introduction

Influenza causes great economic damage each year due to lost productivity and associated medical treatment, as well as indirect costs of preventative measures [1]. The 2009 pandemic reinforced the idea put forth by Glezen that, “The fires of the epidemic are fed by healthy, susceptible school children” [2]. While school summer holiday apparently helped reduce influenza transmission after the initial pandemic wave [3, 4], school reopening dates during the fall of 2009 in the United States coincided with local acceleration of transmission that resulted in a second pandemic wave [5–8]. Numerous reports document the central role of school-aged children in spreading influenza [9, 10]. Children experience higher rates of infection [11], shed influenza virus for approximately twice as long as adults [12] and are thought to have much higher rates of contacts than the rest of the population [13, 14].

Mixing patterns among school children likely contribute to increased transmission of influenza as well as other acute respiratory infections [15–17]. Key elements in characterizing the speed and extent of infectious disease spread [18, 19] are mixing rates and patterns of encounters among school students during normal school times, and during planned and unplanned class and school closures [20]. The statistical properties of social interaction, as characterized by social networks, are crucial in determining patterns of epidemic spread. Knowing the structure of social contact networks enables us to test and assess the effect of different interventions that may change the dynamics of epidemics. In this study, we address the limitation of focusing on only one type or level of school in our understanding of school-based mixing patterns by collecting and analyzing contact patterns with the same technology and setup at schools of different levels and types including public and charter (publicly-funded) elementary, middle, and high schools in the United States.

Recent advances in microelectromechanical systems technology (MEMS), wireless communications, and digital electronics have enabled the development of low-cost, low-power, multifunctional sensor nodes (also known as sensor motes or, simply, motes) that can measure proximity between devices over time [21, 22]. Here we report a network analysis of proximal interactions recorded using motes during the 2012–2013 school year in eight K-12 schools, which includes kindergarten and the 1^st^ through the 12^th^ grades, in greater area of Pittsburgh, Pennsylvania.

## Methods

The social mixing and respiratory transmission (SMART) project was conducted in eight schools, including grades K-12 in two school districts, between October 2011 and April 2012. Schools from two school districts were included: a public school district and a group of charter schools in Western Pennsylvania (both within the Pittsburgh standard metropolitan statistical area [SMSA]). The local government funds both public and charter schools and while public schools are operated by the government charter schools are operated independently by not-for-profit organizations. Parents were offered the opportunity for their students to opt out of the study; students could also refuse to participate. Average opt-out proportion was 7% in schools.

Students in each of the eight schools were assigned a single mote on the deployment day. Sensor network deployment details are shown in Table 1 including number of motes deployed, deployment day of the week and class sizes. We worked closely with school officials to select the mote deployment date, a “typical” day without school testing or school-wide special activities. We distributed motes to teachers and other staff members as well but these data are not included in this analysis. About 500 sensor motes were provided in plastic pouches with lanyards, and students were instructed to carry them around their necks. Some additional stationary sensor motes were deployed throughout the school in classrooms and common areas to provide time synchronization for all motes and determine the spatial location of contacts. In each mote deployment, 1–2 stationary motes were used in each classroom and 1–5 motes were used for larger rooms such as cafeterias or gyms. The deployment durations varied slightly according to school schedules. In general, the motes were distributed before the first class (between 8am-9am) and collected immediately following the last class (between 2:30pm-3:30pm) on the same day. Over multiple deployments, in total, 1,828 students and 116 teachers and staff wore motes, and 232 motes were deployed at fixed locations within the schools (stationary motes). We labeled each school depending on the school district type and level, i.e., a label X-Y in which X denotes the school district (P for public and C for charter) and Y denotes the school level (ES for elementary school, EM for elementary-middle school, MS for middle school, and HS for high school). In each deployment, we covered the entire student population, except in one of the middle schools (P-MS2) and one of the high schools (P-HS) due to large student populations. To remedy this, for P-MS2 and P-HS, we randomly selected classrooms from different grades.

**Table 1:** List of schools along with the numbers of motes used for students, staff members, and rooms.

School	Day	Total	Stat.	Staff	Students	K	1	2	3	4	5	6	7	8	9	10	11	12
P-ES	Mon	184	29	14	141	27	31	21	32	30	-	-	-	-	-	-	-	-
C-ES	Mon	209	21	17	171	30	30	24	14	34	39	-	-	-	-	-	-	-
C-EM1	Mon	296	32	13	251	31	33	16	34	29	35	17	20	36	-	-	-	-
C-EM2	Wed	389	38	24	327	34	35	37	36	40	33	43	37	32	-	-	-	-
P-MS1	Wed	335	22	11	302	-	-	-	-	-	152	150	-	-	-	-	-	-
P-MS2	Wed	211	38	4	169	-	-	-	-	-	-	-	101	68	-	-	-	-
C-HS	Mon	190	18	18	154	-	-	-	-	-	-	-	-	-	74	67	13	-
P-HS	Tue	362	34	15	313	-	-	-	-	-	-	-	-	-	52	87	88	86

Stat.: Number of stationary motes

K: Kindergarten

1–12: Grades 1 through 12

C: Charter school

P: Public school

ES: Elementary school

EM: Elementary-middle school

MS: Middle school

HS: High school

**Fig 1:**
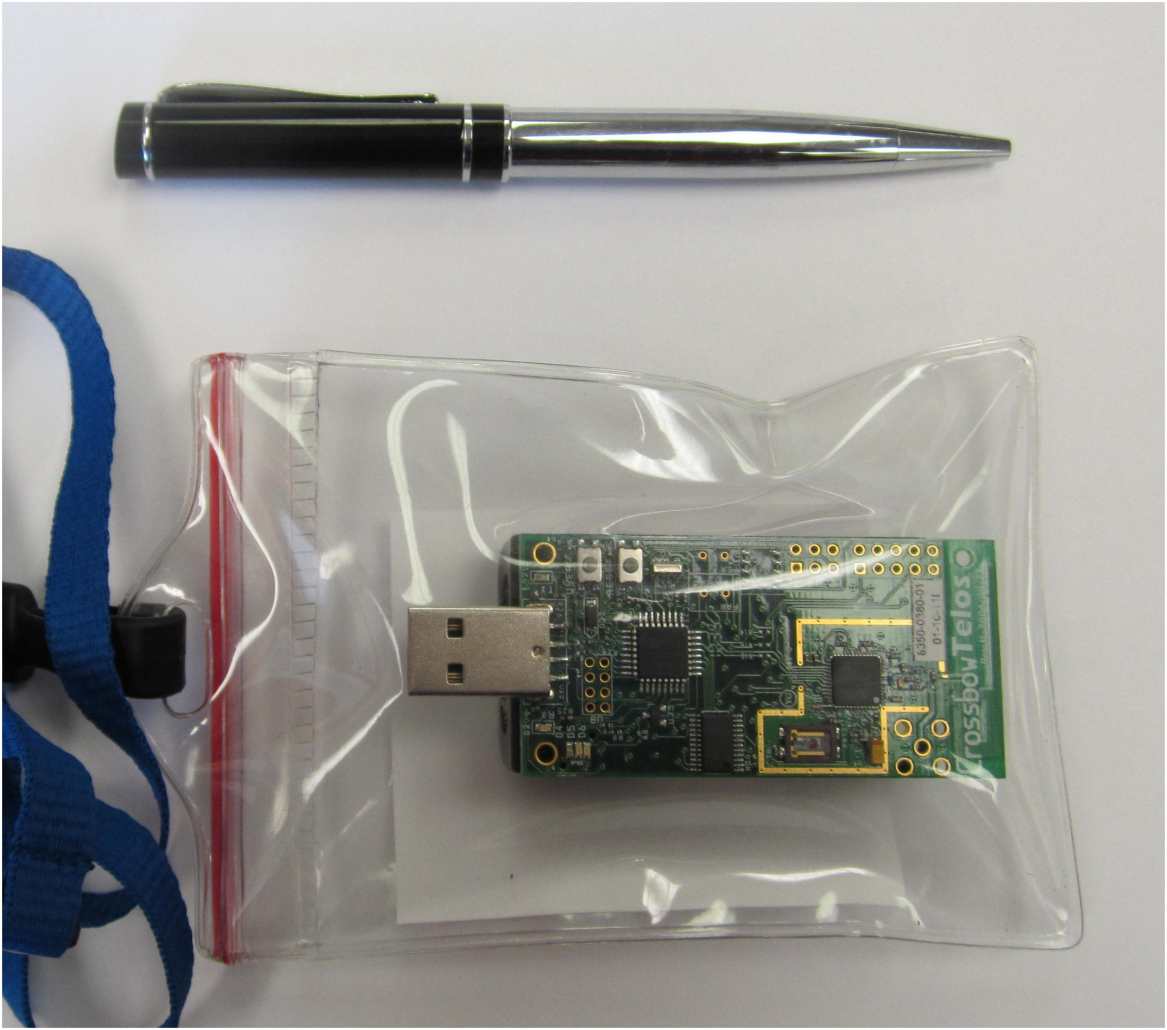
TelosB sensor mote used in the study.

We used TelosB wireless sensor motes manufactured by Memsic Inc. [23] in this study. The size of a rectangular mote is similar to the size of its battery pack, which holds two AA batteries (shown in Fig 1). TelosB sensors utilize an IEEE 802.15.4-compliant radio frequency (RF) transceiver, a 2.4 GHz globally competitive ISM band, and a 1 MB external flash memory for logging contacts.

The sensor motes were programmed in NesC language [24] to transmit a beacon every 20 seconds and listen for other motes’ beacons. Whenever a mote detected another mote, it recorded its unique mote ID, the current time, and the radio signal strength indicator (RSSI). Signal strength provides a measure of proximity of the sensor motes, hence the individuals. An initial pilot investigation found that the signal strength between two motes dropped to about -80 db when they are face-to-face and approximately 3 meters away from each other; this distance can be assumed to be of high relevance to influenza transmission when one considers large droplets from a strong sneeze and enables us to compare our results with the literature [25, 26]. The proximity metric, as measured by signal strength, depends on many factors, including line of sight and the presence of obstructions. In this analysis, we measured uninterrupted continuous interactions among the students, which we call “encounters”, then we accumulated encounters for the deployment day to obtain “cumulative contact” or just “contact” and created student contact networks with weights proportional to the total number mutual recordings. Each recording is assumed to correspond to a continuous 20-second contact between the students. For example, x recordings between a pair of students denote x/3 minutes of aggregated contact time between them, not necessarily consecutive, during that day.

One minor problem with motes we used was the occasional data corruptions in the flash memories due to technical failures and human interventions such as taking out and putting back the batteries. We observed this data corruption problem in about 18% of the motes. We resolved most of the corrupted data issues by reading the memory in the raw format and putting in offsets at places where the corruption started and ended, reducing the data loss to about 5% for these problematic motes.

The study was reviewed and approved by institutional review board (IRB) of the University of Pittsburgh (IRB# REN15020012 / PRO11120186), and under the US Centers for Disease Control and Prevention IRB authorization agreement. In addition, the IRBs of the two universities that collaborated on this study, the Johns Hopkins Bloomberg School of Public Health and the University of Liverpool, also reviewed and approved the study protocol. The demographics of the school populations is slightly different from that of the Pittsburgh Standard Metropolitan Statistical Area (population around 2.6 million): 89.8% Caucasian, 7.7% African-American, 1.1% Asian, and 0.7% Hispanic [27], whereas the school population is 70.5% Caucasian, 25.8% African-American, and 0.9% Asian, reflecting a more urban population.

### Network Analysis

We analyzed network properties, including degree, strength, and, density in order to assess the overall connectivity of the network, as well as to compare different contact networks to each other [28–30]. Degree is the number of contacts accumulated during the deployment period by the students. Strength of a student is the total time he/she spent with his/her contacts during the deployment. Density is the ratio of the number of contacts present in the network and the maximum number of contacts possible. We also calculated the clustering coefficient [31]. Clustering coefficient (also known as local density) is a measure of how much the contacts of a student have contacts with each other and can mathematically be defined as the ratio of the number of contacts a student has and the maximum number of connections among these contacts. We used Igraph [32] library for network analysis and Pajek [33] for network visualizations plotted using a force-based algorithm [34].

In networks where individuals are connected through co-location such as school contact networks we study here, overlap of contacts of students can be used in assessing the strongly formed clusters of students in addition to clustering coefficient. We use a definition of overlap based on common contacts between two students that can be described as the intersection of contact sets of the students [35], O_ij_ = n_ij_/(k_i_-1+k_j_-1-n_ij_), where n_ij_ is the number of common contacts between student i and j, and k_i_ (k_j_) is the degree of student i (j). The behavior of the overlap ratio of two students as a function of the weight of the edge between them (the duration of the contact) is a useful notion to understand the clustering of the students with their short or long-duration contacts.

In order to measure how well a school contact network can be divided into grades or classrooms, we computed modularities for communities defined by either the grades or the classrooms and compared their values. A community is a strongly connected set of nodes, i.e., individuals tagged by motes that is sparsely linked to the remaining network. If the network is divided (fragmented) into communities, such that the number and weight of the contacts between the communities are small and the contacts between the nodes in the same community are large in number and weight, then the network has a high modularity for this specific division. The structure of communities in a network can be considered a medium-level topological organization as opposed to local and global structures [36]. The technical definition of modularity [37] is based on the idea that a random network is not expected to have a modular community structure, so the possible existence of communities is revealed by the comparison between the actual density of contacts in a community and the density one would expect to have in the community if the network nodes were attached regardless of community structure.

## Results

Basic network measurements for school contact networks are presented in Table 2, including number of students (n), density of the network (δ), average degree (number of contacts, d), average time per contact in minutes or strength (s), and average clustering coefficient (CC). Some network measurements are very sensitive to high number of short-duration contacts or cannot use weights, for that reason, we omitted contacts with duration less than 5 minutes for better description of network measurement statistics and for visualization. In other words, we assume that two students have a contact if their total interaction time on the deployment day is greater than 5 minutes. The network densities varied greatly from school to school, with values ranging from 0.096 for P-MS1 to 0.345 for C-HS. The densest network was C-HS, a small school in a relatively small space. The other high school (P-HS), however, is a very low-density (0.114) network with more than 1,500 students. We had to deploy our motes in a subset of randomly selected classrooms, affecting average degree and average contact duration. The format of the raw mote data and additional information can be found in S1 Text. Also, all the network files used in this study are available in S1 Data in various network file formats.

**Table 2:** Contact network measurements and their standard deviations (and standard errors) for school contact networks*.

School	n	δ	d	s	CC
P-ES	141	0.189	27.6 ± 9.1 (0.9)	38.3 ± 14.8 (1.4)	0.81 ± 0.12 (0.02)
C-ES	171	0.147	25.6 ± 10.3 (0.8)	41.9 ± 10.3 (0.9)	0.82 ± 0.14 (0.01)
C-EM1	251	0.108	29.1 ± 10.1 (0.8)	43.3 ± 15.6 (1.2)	0.76 ± 0.14 (0.02)
C-EM2	327	0.116	38.0 ± 13.3 (0.8)	44.9 ± 18.8 (1.1)	0.76 ± 0.13 (0.01)
P-MS1	302	0.096	30.0 ± 9.5 (0.6)	52.8 ± 20.0 (1.3)	0.72 ± 0.18 (0.01)
P-MS2	169	0.302	52.9 ± 19.3 (1.7)	27.4 ± 6.2 (0.6)	0.71 ± 0.09 (0.01)
C-HS	154	0.345	56.1 ± 15.5 (1.6)	21.7 ± 3.9 (0.5)	0.56 ± 0.06 (0.01)
P-HS	314	0.114	35.9 ± 11.7 (0.6)	17.8 ± 4.4 (0.3)	0.41 ± 0.15 (0.01)

* The contacts with total duration less than 5 minutes are ignored

n: number of students

δ: density of the network

d: average degree (number of contacts)

s: strength (average duration per contact) in minutes

CC: average clustering coefficient

C: Charter school

P: Public school

ES: Elementary school

EM: Elementary-middle school

MS: Middle school

HS: High school

Across all schools included in this analysis, we found that students had an average of 26–56 contacts lasting cumulatively 5 minutes or more during a typical school day. The highest average number of contacts was observed at C-HS (about 56) due to a well-mixed contact network. Although we had a partial deployment at P-HS, the degree was around 36, greater than all the elementary schools and one of the elementary-middle schools. In general, the lower-level schools have low degrees but longer average contact duration, demonstrated by s range in Table 2. Clustering coefficients varied from 0.41 to 0.82 in these contact networks. The high value of clustering coefficient in elementary and elementary-middle schools (0.71–0.82) may be due to similar schedules among students in the same grade.

**Fig 2:**
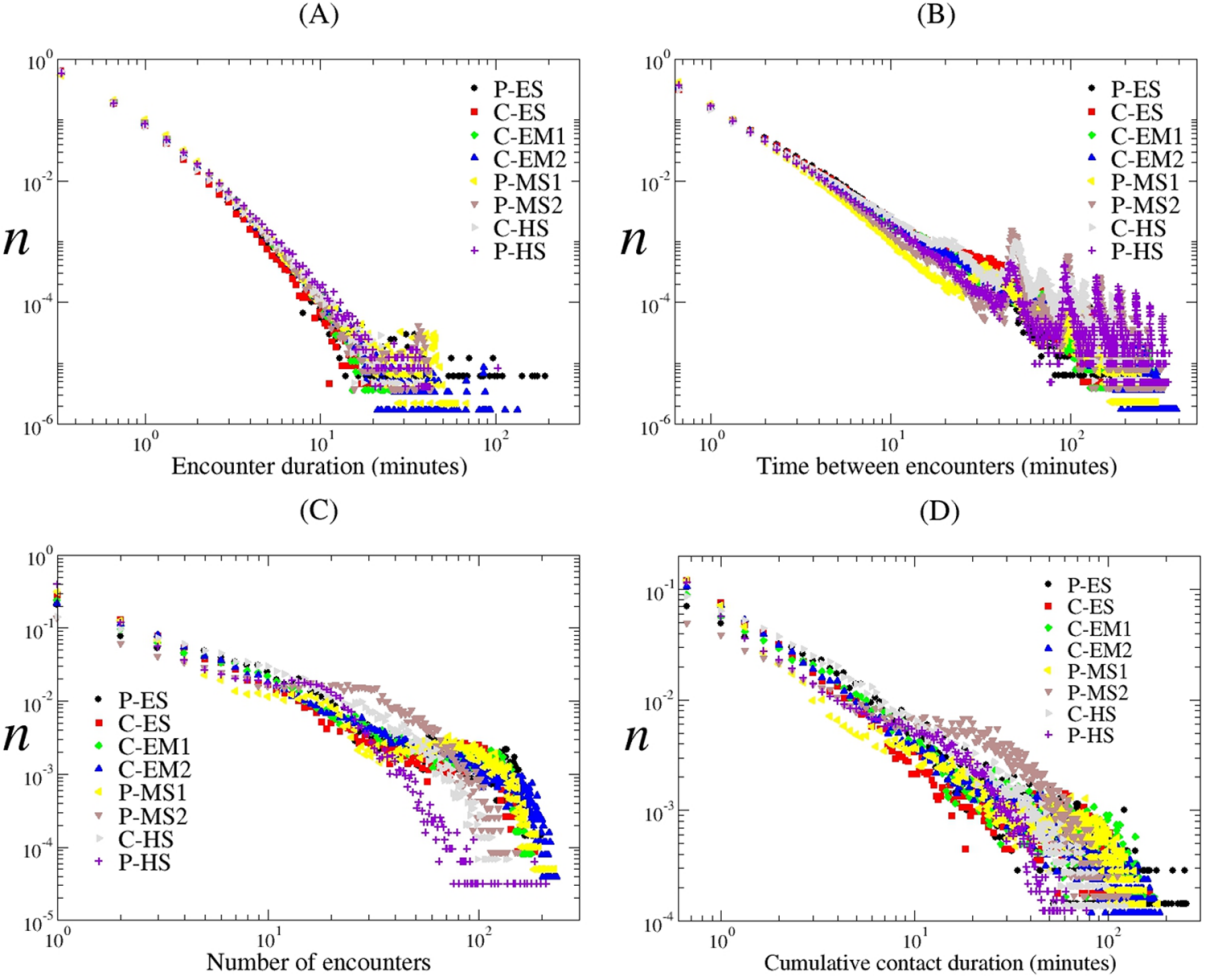
Encounter and contact duration distributions. *n*: relative frequency; C: Charter school; P: Public school; ES: Elementary school; EM: Elementary-middle school; MS: Middle school; HS: High school.

Encounter and cumulative contact-duration distributions showed similar characteristics at schools of different levels. The panels in Fig 2 show the distribution (relative frequency) of the encounter durations (A), time between encounters (B) and number of encounters (C) of the same pair of students, and cumulative contact durations (D). All these distributions follow a power law, as observed in other school contact network studies [26, 38]. One can observe from Fig 2A–2C that the encounters are very dynamic, namely, most of them are short and frequent, a natural result of long-tailed power-law distributions.

The distribution of cumulative contact durations as seen in Fig 2(D) shows that the proportion of all contacts that are ≤ 1 minute are P-ES (31%), C-ES (47%), C-EM1 (36%), C-EM2 (37%), P-MS1 (47%), P-MS2 (21%), C-HS (27%), and P-HS (52%). The proportions of contacts ≤ 5 minutes are P-ES (60%), C-ES (72%), C-EM1 (61%), C-EM2 (64%), P-MS1 (64%), P-MS2 (39%), C-HS (60%), and P-HS (71%). Although 5 minutes is an arbitrary duration, it can be used to separate a weak contact from a strong one [39]. By using a method described in [40], we fitted a power-law function P(w) ~ w^β^ to contact duration distributions (Fig 2D) and found β values ranging from -0.9 to -1.4. This power-law behavior of contact-duration distributions was observed in other school contact studies [26, 38]. Contact duration distributions also show some peculiar effects due mostly to school schedule. Short-duration contacts (1–10 minutes) appear to be distributed in a power-law fashion for only about one order of magnitude, and medium-duration contacts (10–30 minutes) show bumps in distribution in both middle schools and P-HS. Short-duration contacts can be considered as occasional contacts between students from different grades or classrooms during breaks and lunchtime. Medium-duration contacts are usually in-class contacts sitting at a distance in the same classroom. Long-duration contacts (greater than 30 minutes) among students (shown at the tail of the distribution) are observed relatively less frequently with exponentially decaying probability: P-ES (16.1%), C-ES (13.5%), C-EM1 (17.6%), C-EM2 (15.9%), P-MS1 (18.6%), P-MS2 (21.2%), C-HS (8.9%), and P-HS (3.4%). Although the distributions of cumulative contact durations follow a power law, the signal strengths of all encounters are distributed exponentially (results are not shown). One can argue that the exponentially distributed signal strengths make the choice of threshold relatively arbitrary, not epidemiologically but in terms of the networks generated based on these values.

**Fig 3:**
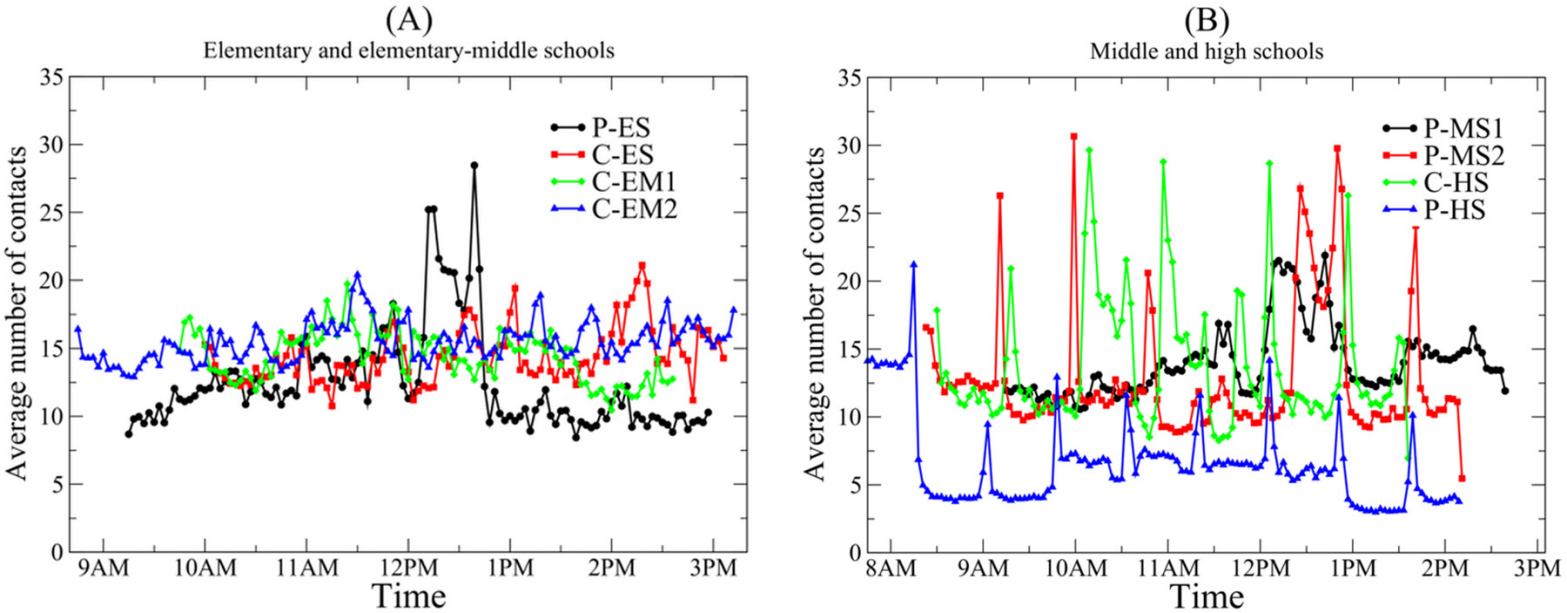
Average number of contacts (degree) for 3-minute intervals for the duration of the deployment. A) Elementary and elementary-middle schools B) Middle and high schools. C: Charter school; P: Public school; ES: Elementary school; EM: Elementary-middle school; MS: Middle school; HS: High school.

We characterized changes in contacts during each day. We calculated the average degree (number of contacts) per student in 3-minute intervals on deployment day (Fig 3). In middle schools and high schools, the average number of contacts is low when students are in their classrooms and high when students have the chance to contact more schoolmates, such as during breaks and lunchtime. The graph in Fig 3A shows the average degree at 3-minute intervals in two elementary schools and two elementary-middle schools. The starting times are different because the deployment and/or the schools started at different times. The class breaks were vaguely visible and the lunchtime overcrowding in the cafeterias was not observable at all, except for at P-ES. In these elementary and elementary-middle schools, lunchtime is relatively quiet because each grade eats lunch together with another grade at different time slots from 11am to 1pm, creating less dense contact activity in the cafeterias during lunchtime. P-ES, however, has a very high average degree during lunchtime compared to class times because of a physically small cafeteria and lunchtimes and recess that are concentrated between 11:45am and 12:45pm; higher-level schools, such as middle schools and high schools, are shown in Fig 3B. These schools exhibit very pronounced differences in average degree during class breaks. All of the schools, except one of the middle schools, P-MS1, showed increase in average number of contacts during class break. Although P-MS1 has no visible differences in number of contacts during breaks, it has a shorter lunch period and relatively smaller cafeteria, creating a higher average number of contacts during that time.

**Fig 4:**
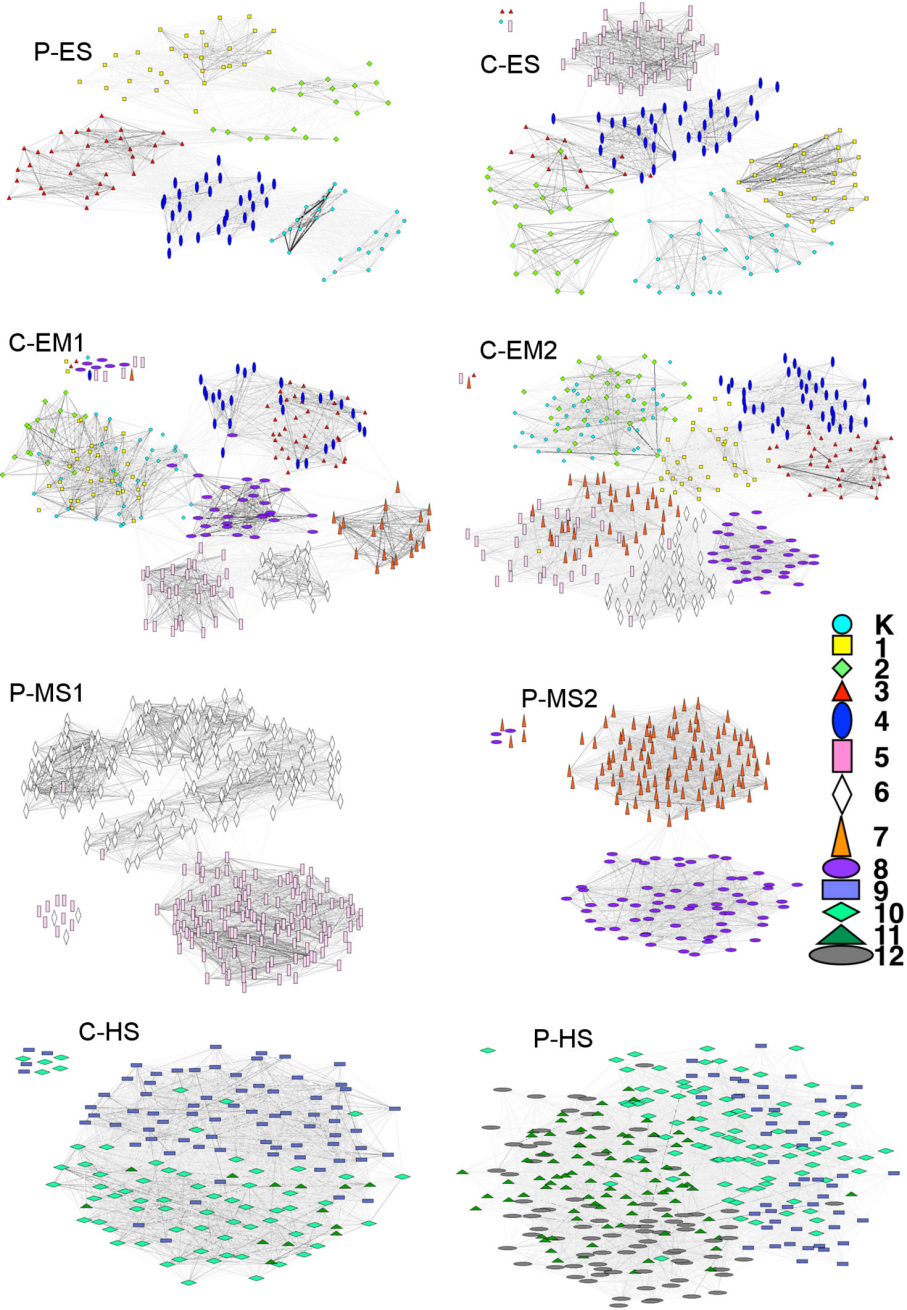
School contact network visualizations*. * The darkness of the lines is proportional to contact durations and the shape and color of the nodes show the grade of the student. The contacts with total duration less than 5 minutes are not shown. K: Kindergarten; 1–12: Grades 1 through 12; C: Charter school; P: Public school; ES: Elementary school; EM: Elementary-middle school; MS: Middle school; HS: High school.

Network visualizations in Fig 4 show the students as nodes and the contacts among them as lines if the total contact duration is greater than 5 minutes. Students in the same grade were identified in visibly separated groups in all networks except high schools. In the high schools, the students are connected to other students in different grades, making it difficult to visually identify the grades or classrooms.

**Fig 5:**
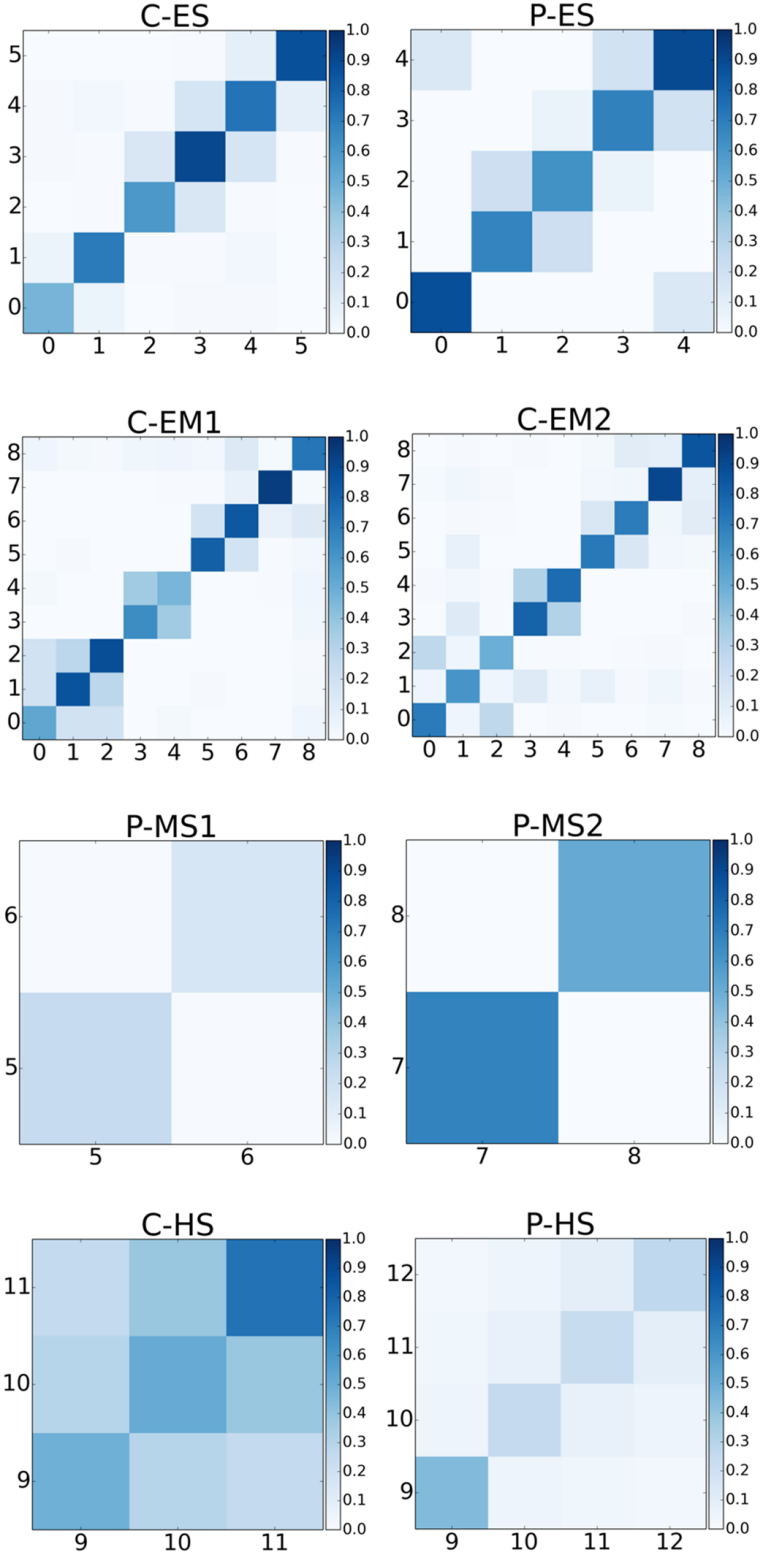
Contact matrices among the grades for each school*. * Each cell represents number of contacts between the grades per pair of students. The contacts with total duration less than 5 minutes are ignored. Each column and row corresponds to a grade and 0 is used for Kindergarten. C: Charter school; P: Public school; ES: Elementary school; EM: Elementary-middle school; MS: Middle school; HS: High school.

The heat maps can be used to visually display contact matrices, i.e., inter-grade and intra-grade mixing patterns in each school. Fig 5 shows the average number of contacts per pair of students among grades for each school as a heat map. A greater number of proximal contacts occur among students in the same grade. In elementary and elementary-middle schools, a relatively large number of contacts can occur between some pairs of grades due to common lunch schedules (e.g., 1^st^, and 2^nd^ grades in C-ES and P-ES).

In both of the public middle schools we studied, each grade is not only physically separated but also has a schedule that does not overlap other grades, making most student contacts with students in the same grade. However, in high schools, due to very diverse student-centered schedules as opposed to grade-specific schedules in lower-level schools, contacts are distributed throughout all grades.

**Fig 6:**
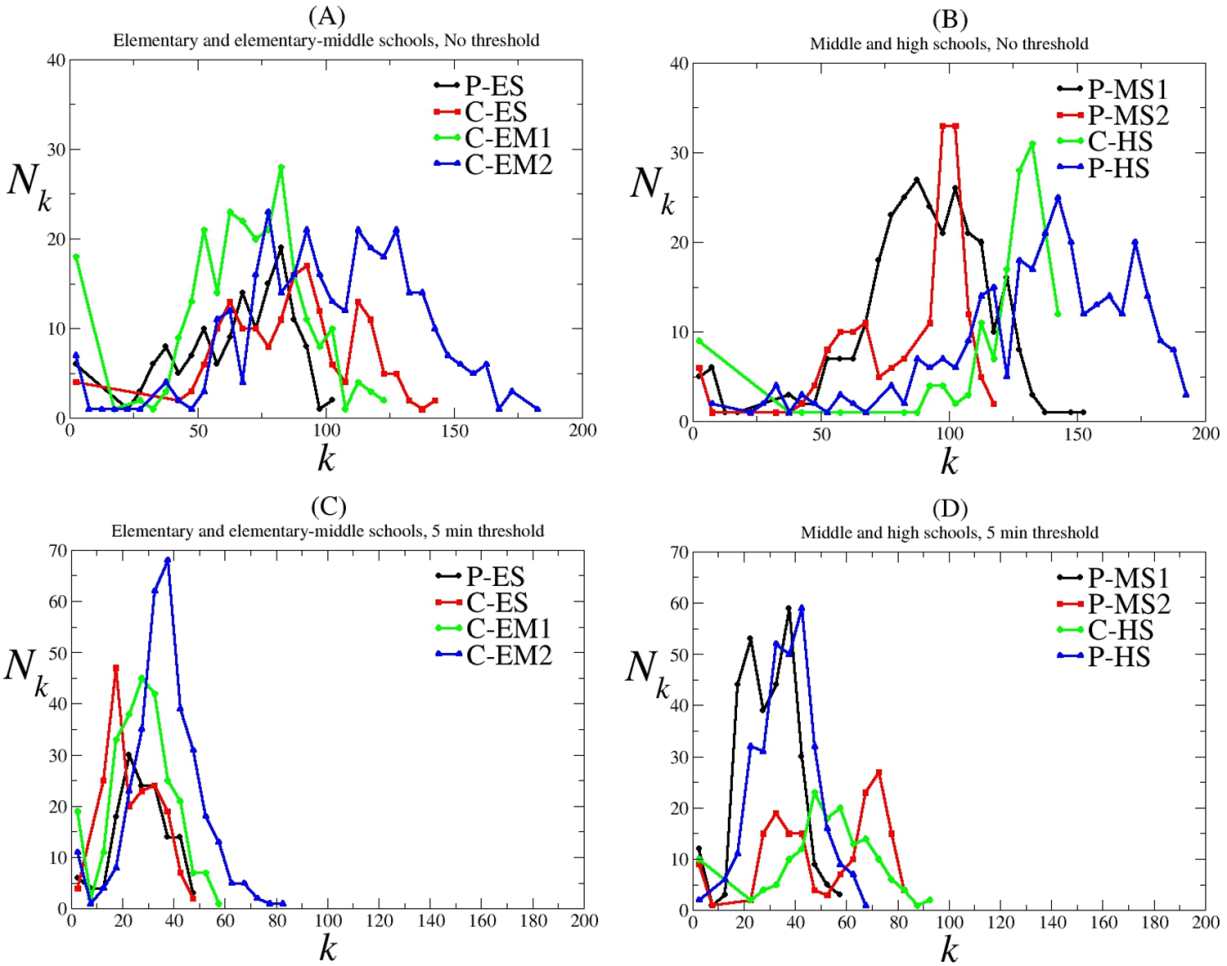
Degree distributions of contact networks. (A) Elementary and elementary-middle schools, no threshold (B) Middle and high schools, no threshold (C) Elementary and elementary-middle schools, 5 min threshold (D) Middle and high schools, 5 min threshold. *k*: the number of contacts (degree); *N*_*k*_: the number of students with *k* contacts; C: Charter school; P: Public school; ES: Elementary school; EM: Elementary-middle school; MS: Middle school; HS: High school.

We explored the impact of placing thresholds on the duration of proximal contacts required to define a contact between two individuals on degree distributions of the networks. Degree distributions of contact networks can be seen in Fig 6. In general, degree distributions are binomial-like and centered on the average degree values (listed in Table 2).

**Fig 7:**
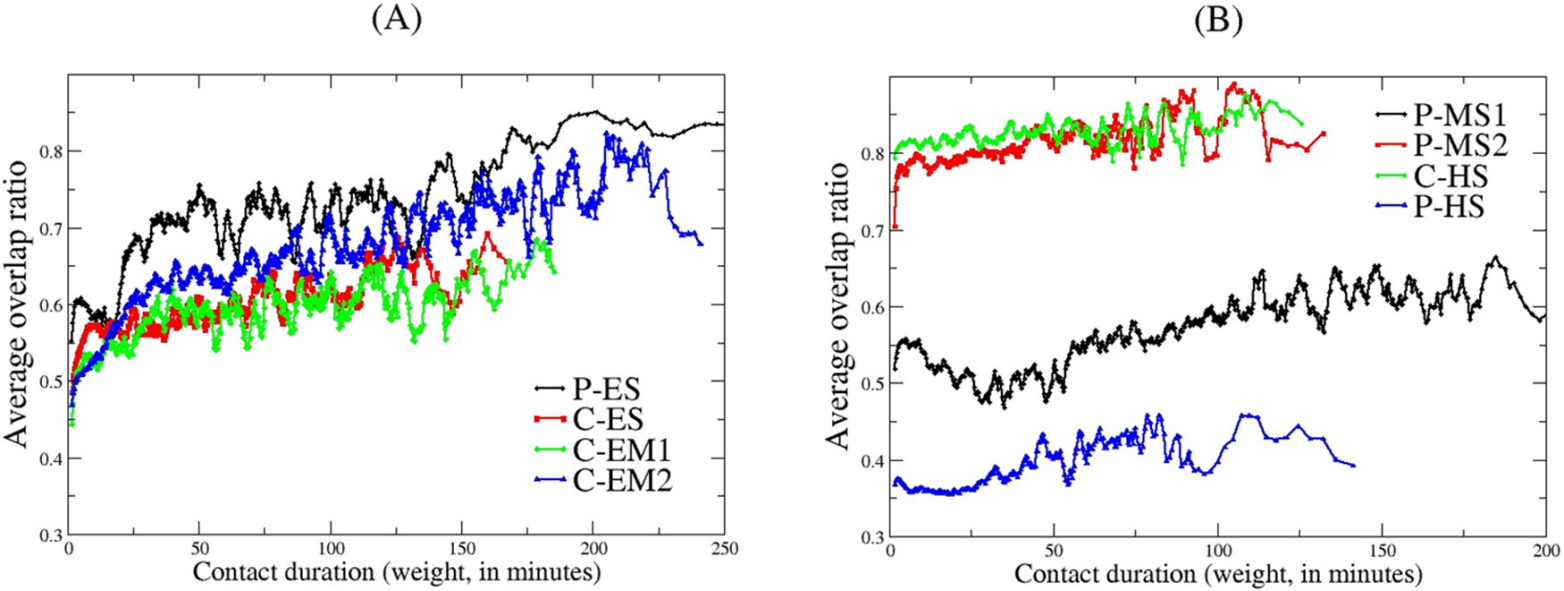
Average overlap ratio versus contact duration. (A) Elementary and elementary-middle schools (B) Middle and high schools. C: Charter school; P: Public school; ES: Elementary school; EM: Elementary-middle school; MS: Middle school; HS: High school.

Average overlap ratios as a function of contact duration (link weight) for each school is shown in Fig 7, grouped as (A) elementary and elementary-middle schools and (B) middle and high schools. Average overlap ratio is typically low for short-duration contacts, whereas, long-duration contacts have higher overlap ratio because they are usually among classmates who are in contact almost the whole day forming strongly connected clique-like sub-networks. In all schools average overlap ratio slowly increases with contact duration except with some fluctuations for short-duration contacts. We observed the least average overlap ratio in P-HS, the largest school we had to do a partial deployment, and on the other hand the middle schools have the highest average overlap ratio.

We used modularity scores (shown in Table 3) to measure the community structure of these networks. As expected, the grade and classroom divisions have relatively high modularity in elementary and elementary-middle schools (0.54–0.75). In these schools, all students have fixed schedules (i.e., the same courses in the same time slots in the same classrooms); however, classroom and grade divisions are not perfect (modularity is not unity) due to occasional contact among students from different grades and classrooms.

**Table 3:** Modularities of the networks based on grades and classrooms.

School	Grade Modularity	Classroom Modularity
P-ES	0.68	0.67
C-ES	0.71	0.71
C-EM1	0.73	0.67
C-EM2	0.75	0.54
P-MS1	0.48	0.77
P-MS2	0.35	0.09
C-HS	0.22	0.11
P-HS	0.36	0.32

C: Charter school

P: Public school

ES: Elementary school

EM: Elementary-middle school

MS: Middle school

HS: High school

The network visualizations (shown in Fig 4) help guide our understanding of modularity values. In elementary and elementary-middle schools, the grades and classrooms are visually distinguishable: at the grade level, the middle schools are not as modular as elementary and elementary-middle schools and at the classroom level, middle schools have very different modularities. P-MS1 have 0.77 (the highest among all modularities we measured) and P-MS2 have 0.09 (the lowest). P-MS1 have a very visible classroom structure for 6^th^ graders, increasing the modularity; however, the 5^th^ graders form a well-connected single community due to common activities. At P-MS2, only a portion of the student population has been observed due to the size of the school: one group among 7^th^ graders and one among 8^th^ graders. These two groups are clearly visible in Fig 4 as two separate communities connected only by occasional short-duration contacts; the classrooms were not discernable. The high schools have very low modularity at both the grade and classroom level. The mixed schedules of students in these schools (i.e., students from different grades take the same classes) decreases the modularity based on grades or classrooms; C-HS has the lowest modularity score based on grade (shown in Fig 4 with faintly discernable grades).

**Fig 8:**
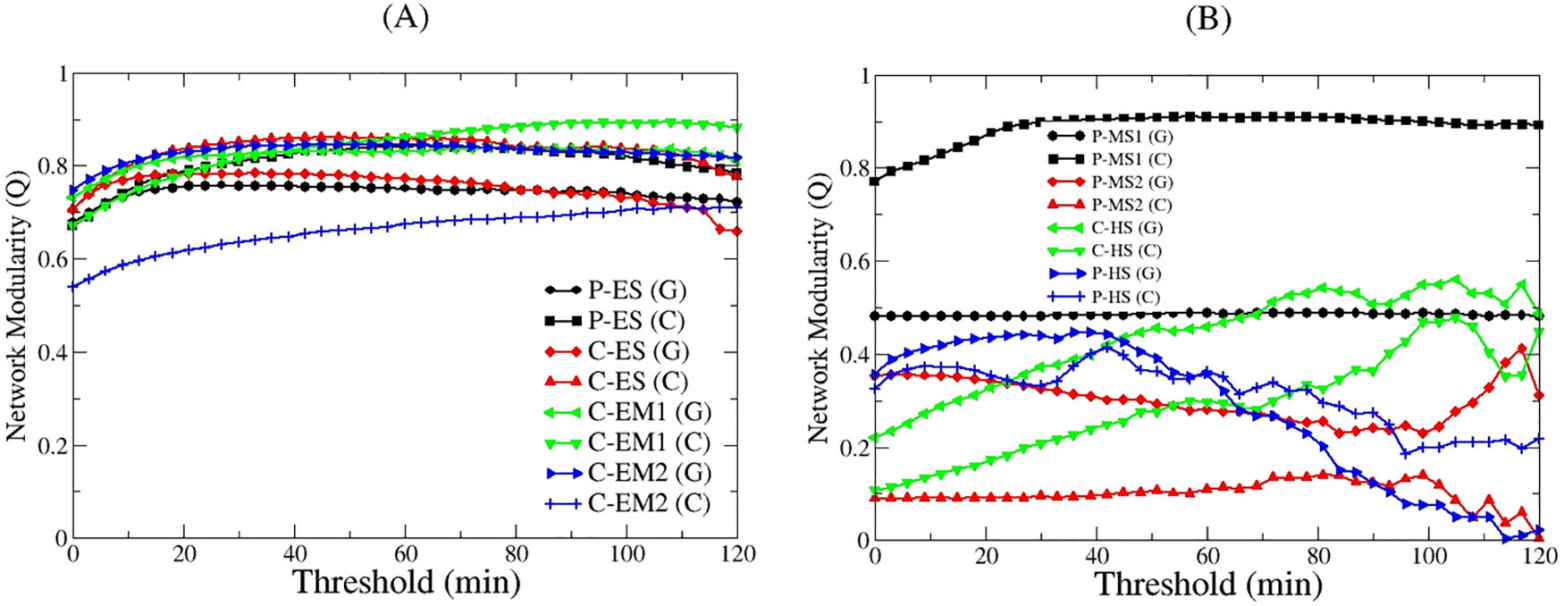
Network modularity versus threshold*. (A) Elementary and elementary-middle schools (B) Middle and high schools. * The contacts with total duration less than threshold are ignored. (G): grade-based modularity; (C): classroom-based modularity; C: Charter school; P: Public school; ES: Elementary school; EM: Elementary-middle school; MS: Middle school; HS: High school.

In order to assess the effect of the short-duration contacts on modularity scores in these schools, we examined the modularity score against the threshold (shown in Fig 8 with increments of 10 readings corresponding to 3 minutes and 20 seconds), allowing some insight into the dynamics of the modularity as we change the threshold. In general, having weak links decreases modularity; in other words, the modularity increases as we increase the threshold by deleting the contacts with duration less than the threshold. After reaching a certain threshold, removing links weakens the modular structure of the network and, thus, causing a slow decrease. This threshold appears to be corresponding to a value that roughly separates intra-classroom contacts from inter-classroom contacts. Modularity scores for very high thresholds are not reliable because of an insufficient number of contacts in the network. This generic behavior is observed in our elementary and elementary-middle schools (shown in Fig 8A); however, a very peculiar behavior of modularity is seen in higher-level schools. Fig 8B shows the modularity scores based on grades and classrooms versus threshold for middle and high schools. Grade-based modularity stayed almost constant against threshold for P-MS1; classroom-based modularity for the same school have a generic behavior (i.e., first increases then decreases very slowly). P-MS1’s grade-community structure was already very prominent, so applying a threshold did not really change the modularity; classroom communities are weakly connected, so removing them increases the modularity. At P-MS2, the classroom-based modularity stayed constant for a wide range of threshold values but, interestingly, grade-based modularity decreased with threshold. The reason for this behavior is that the classrooms are not easily separable from each other (hence, low classroom-based modularity to begin with), and already separated grade communities weaken as contacts are deleted. The high schools exhibited generic behavior for modularities, with fast increases for low values of threshold and fluctuations afterward due to highly connected grades and classrooms separated from each other with increasing threshold, as well as removing any remaining long-duration contacts one by one.

## Discussion

This study addresses a number of gaps in our understanding of social mixing patterns of school-aged children attending U.S. elementary, middle and high schools. Our analysis has shown that contacts among these age groups differ along multiple dimensions, including the mean number of proximal contacts during school, the duration of these contacts, and the clustering and modularity of proximity-based contacts. Our results suggest that lower-level schools, such as elementary and elementary-middle schools, have very typical contact patterns due to fixed schedules and can be modeled as fully mixed classrooms with weak inter-grade interactions. The students in middle schools and high schools have relatively free schedules and are relatively well mixed across grades and classrooms.

The estimates we derived from the contact data, such as the number and diversity of contacts and their durations and locations, could play an important role in determining the extent and speed of a respiratory infection transmission. In well-mixed networks with typically high average degree, for example, the transmission can occur more rapidly, resulting in higher attack rates, assuming a fully susceptible population [41]. Although younger children are at greater risk of influenza and other acute respiratory infections, weak links between communities (i.e., high modularity) within a network, as observed at elementary schools, could limit or slow infectious disease transmission between these communities, resulting in a lower overall school attack rate. Lower-level schools function as a set of loosely connected classrooms and grades, whereas high schools have social contacts that are more mixed by grade, creating a more tightly connected network across all grades that is not so different than a completely random network for modeling purposes.

In general, high modularity in social networks implies quasi-isolated groups and in case of low-vaccination coverage at the group level, they have the potential to create locally accelerated influenza infections [42]. The fact that the weak links (short-duration contacts) among the students in different classrooms or grades and strong links (long-duration contacts) among the students in the same classroom or grade attest to the observation that weak links may play an important role in network such as carrying the virus from one group to another (Granovetter’s “strength of weak ties” theory [43]). Mathematical modeling could help further address these hypotheses.

Our findings also highlight the role of physical school floor plans and inter-grade activities on social contact mixing patterns. Cafeteria size and the layout of the hallways connecting classrooms appear to impact the number and duration of school contacts. These observations could be helpful in modeling interventions to reduce in-school social mixing and assessing impact on influenza transmission. On the other hand, public district schools and charter schools were similar in terms of network measurements. Although these schools are administered using different business models and they are also different in size (typically public district schools are larger and more crowded), their networks are similar not only visually but also in degree distributions and other connectivity metrics as well as modularity.

The POLYMOD study [13] quantified mixing patterns for eight European countries using age- and gender-representative samplings of the populations and observed strong assortative mixing of age groups and particularly high rates of assortative mixing among school-aged children. Although the POLYMOD study found relatively few differences in mixing patterns across these countries, it is unknown if the information generated is appropriate for public health purposes within the United States, particularly in school-aged children, given the differences not only in educational institutions but also in urban culture of space usage. In addition, respondents provided information about a single day only, no higher-level network information was collected, and information was gathered from the total range of respondents during a more than 1-year period. Since our study focused on in-school contacts only, our age-specific contact matrices were more diagonal than those reported in POLYMOD.

Another study of school-based contacts was carried out in a US high school consisting of 800 students (grades 9–12), teachers, and staff using motes [26]. They found that a social network formed by connecting individuals who were in close contact (3 m), resulted in a very dense network (about 750,000 close contacts), with a low mean network distance between individuals and a relatively homogeneous connectivity distribution along with high clustering. In a subsequent study same as above [44], similar sensor motes were deployed in another high school (715 students) on three different school days. Similar contact network properties were observed, including high density and clustering, as well as high modularity. Our study included a much larger student population across several different types of schools and grades in urban and suburban settings. In contrast, our study found differences in multiple contact-structure metrics by school and grade range, including higher modularity, clustering, and mean contact duration in lower-level schools compared to higher-level grades. In [26], the authors also found power-law distributed contact and encounter durations as well as another study [45] in which phones with Bluetooth technology was used instead of sensor motes. In [45], the statistical fit of contact duration distributions gave a power-law exponent of -1.33, within the range we found for different schools, -0.9 to -1.4.

An additional study measured face-to-face contact patterns at a distance of 1–1.5 m in a French primary school (232 students aged 6–12 years for 2 days) [38]. The authors calculated that the students spent, on average, three times more time in contact with classmates than with children in other classrooms. In our study, the total contact-duration ratio for students in the same and different grades exhibits great variation (3%-75%), being higher in high schools and lower in other schools. In this study, the authors also presented results on the number of contacts as a function of time of the day, in which class and lunch breaks are quite visible with more students having contacts with each other. Similar phenomena can be observed in our middle and high school contacts whereas the contacts in elementary and elementary/middle schools stay fairly constant throughout the day. The only exception to this is one of our elementary schools in which during lunchtime average number of contacts peaks because all students have lunch and recess at the same time. We also observed a hierarchical block-diagonal structure on the contact matrix similar to the previous study [26]. A third, larger-scale study measured proximities between individuals at a conference and exhibition using radio frequency identification (RFID) tags worn by participants [46]. Similar technology was used in a high school in France over multiple days in two consecutive years [47] and researchers found that intra-class contacts are much stronger than those among classes but the overall network is still structured with visible classes as communities and the overall structure was robust over different days and years. Our high school contact network is relatively more mixed and it is difficult to discern grades as communities. The difference in term of schedules in American and French high schools reveals itself in the structure of contact networks, i.e., the American high school system is more student-based and there is no homeroom for students.

The choice of a threshold corresponding roughly to 3 meters affects the contact network properties we generated but in order to be consistent with the literature using the same sensor technology [26] we used the same threshold value. Since the distributions of the signal strengths follow perfect power laws for all schools, choosing a little larger threshold (corresponding to contact distances less that 3 meters) would yield networks with degree distributions similar to current ones. Further studies using different thresholds could show differences in the overall structure of the contact networks.

In a more recent work [48] the researchers used not only a sensor network but also contact diaries and friendship surveys for contact structure and compared them to each other. Their observations that the contact durations are distributed in a power-law fashion and high number of contacts is present among the students in the same grade as opposed to weak connections among different grades are parallel to ours in this study.

### Limitations

This analysis is subject to a few limitations. First, to prolong battery life, the sensor motes were programmed to wake up every 20 seconds to gather information about other motes. We assume that if two motes have records of each other in two consecutive time ticks, then they have a continuous contact of 20 seconds. Previous contact network studies suggest that most contacts can be captured at an adequate temporal resolution with this assumption [26, 49]. Data corruption we experienced in some motes was resolved by an offsetting procedure that reduced the overall data loss to about 5% for these motes. Also, we lost data from about 5% of our motes completely due to misuse by the students as well as some technical problems with the mote chips, creating some small-scale missing data problems. Overall data loss was estimated to be < 10%.

## Conclusions

Our findings describe the social-contact network characteristics of school-aged children attending elementary, middle, and high schools in Pittsburgh, Pennsylvania. We detected differences in our contact measurements by school level, likely important differences in understanding influenza transmission among school-aged children in the United States. Further research should explore the effect of contact networks on the spread of acute respiratory infectious diseases, such as influenza, in school settings. We hypothesize that schools with high modularity should be able to control the spread of disease by employing social distancing measures to reduce connectedness between smaller clusters of students, which would be most applicable to elementary schools. This research may have a significant impact on school responses to influenza and other acute respiratory infectious diseases, for example, school closure is often mentioned as a pandemic intervention. This work will contribute to further studies to measure the effectiveness of school closures, as well as raise consideration of other strategies, short of full closure, that may be effective.

## Supporting Information

S1 Data Network files used in the study. They do not contain any identifying information. (ZIP)

S1 Text Supplementary information. (DOCX)
