# Supplementary material for: Social Contact Networks and Mixing among Students in K-12 Schools in Pittsburgh, PA
Source: PLoS One. 2016 Mar 15;11(3):e0151139. doi: 10.1371/journal.pone.0151139 (PMC4792376; doi:10.1371/journal.pone.0151139)
Supplement: S1 Text — (DOCX) [file pone.0151139.s002.docx]

**SUPPLEMENTARY INFORMATION for**

**Social Contact Networks and Mixing Among Students in K-12 Schools in Pittsburgh, PA**

Hasan Guclu^*^, Jonathan Read, Charles J. Vukotich Jr, David D. Galloway, Hongjiang Gao, Jeanette J. Rainey, Amra Uzicanin, Shanta M. Zimmer, Derek A. T. Cummings

^*^ Corresponding Author, Department of Health Policy and Management and Public Health Dynamics Laboratory, Graduate School of Public Health, University of Pittsburgh, Pittsburgh, PA, USA. E-mail: [guclu@pitt.edu](mailto:guclu@pitt.edu). Phone: (412) 624-2178.

**SMART Team**

SMART^[[1]](#footnote-1)^ (Surveillance, Monitoring Absenteeism, Respiratory Transmission in Schools) team includes researchers from Graduate School of Public Health (Pitt Public Health) at the University of Pittsburgh, Pittsburgh Supercomputing Center, Johns Hopkins University, University of Liverpool, and US Centers for Disease Control and Prevention (CDC).

- Pitt Public Health
  - Shanta Zimmer, CoPI
  - Chuck Vukotich, PM
  - Hasan Guclu, CI
  - John Grefenstette
  - David Galloway
  - Kan Li
  - Mary Lou Schweizer, PC
  - Samuel Stebbins (Alum)
  - Jennifer Cousins (Alum)
  - Kathleen Creppage (Alum)
- PSC
  - Shawn Brown, CI
- Johns Hopkins
  - Derek Cummings, CoPI
  - Eva Noble
- Liverpool
  - Jonathan Read, CI
- CDC (funder)
  - Jeannette Rainey
  - Amra Uzicanin
  - Hongjiang Gao
  - Andrew Plummer
  - Andrew Hopkins

**Technical Information About Sensor Motes^[[2]](#footnote-2)^**

MEMSIC’s TelosB Mote (TPR2420) is an open-source low-power (2 AA batteries) platform designed to enable experimentation for the research community. It has USB programming capability, an IEEE 802.15.4 radio with integrated antenna, and 1MB flash memory. The TelosB platform was first developed University of California at Berkeley. It is compatible with the open-source TinyOS distribution^[[3]](#footnote-3)^.

**Raw Mote Data**

At the beginning of the deployment we programmed the motes and gave a unique id number to each and every one of them. During the deployment the motes wake up in every twenty seconds and communicate with all other motes they can and record the id numbers of these motes to their flash memory. At the end of the deployment we downloaded the data on the motes by using their USB communication ports. The resultant files are log files with a format shown in the following figure. This log file belongs to a specific mote (source mote) and each line corresponds to a communication between this mote and one another mote (partner mote). The columns are partner mote id, time tick of the partner mote, signal strength, and time tick of the source mote, respectively. Time ticks are measured in multiple of 20 seconds, hence 3 ticks would make one minute. Also, in some motes it is possible to see some synchronization errors, namely different time ticks for source and partner mote. These discrepancies, although not significantly affecting our analyses, are reduced to minimum by assigning some specific motes (the motes with id numbers at the nine thousands) synchronizer roles. These are typically the stationary motes we put into each room and open space at schools. Whenever a mote communicates with the synchronizer mote it updates its time tick accordingly. In order to reduce the data load on the mote’s processor and memory we discarded all the packets with a signal strength less than the value corresponding to approximately an interaction at 3 meters, namely 220.

**
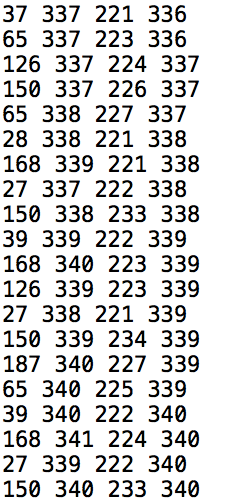
**

In order to accommodate the amount of interaction between the motes (and hence the individuals carrying them) we used a weighted (also known as valued) network model. The social contact network data is obtained by processing these raw files in the following way: The interactions between the motes for the whole deployment (typically from the first class early in the morning to the last afternoon class) are combined together to calculate the amount of interactions in time ticks (20 seconds). We assume that if two motes recorded interactions in two consecutive time ticks, they are in interaction for the whole period in between. This creates a very negligible number of miscounting in the interactions between the motes. Additionally, for this study, we did not consider the interactions between the students and staff members including teachers and administrative/service personnel to be studied later. The network files we generated from the raw data are available within this supplementary information.

**Schedule versus Mote Data**

Many models and simulations of infectious diseases tracking infections in school settings use the schedule of the students based on grade and/or classroom. Typically, it is assumed that the students interact more with their classmates than their schoolmates. The strength of interactions are calculated by using survey-based methods and placed on an interaction matrix as shown as a heat map in the left panel of the following figure for a school with kindergarten to fourth grades.

**
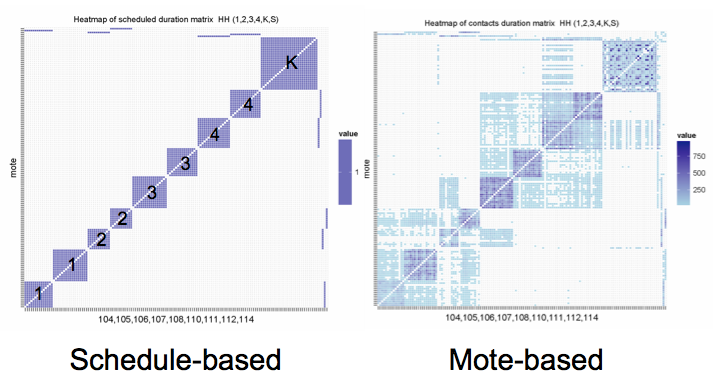
**

**S1 Fig. Comparison of contact matrices for a public elementary school (P-ES) based on motes and schedules.**

One of the major aims of this study is obtaining the interaction matrix in an accurate and realistic way by using motes. After analyzing the social contact network data collected from the motes we obtain an interaction matrix as seen on the right panel of the S1 Fig. This matrix displays some expected characteristics such as the interactions among the students of the same classroom as colored blocks on the diagonal. Another type of interactions is the ones between the students in the same grade but different classrooms. We can also observe contacts between specific grades such as between first and second graders, and between third and fourth graders due to common lunch and recess times, another characteristic that can be found from the schedules but only qualitatively. Some unforeseen interactions also reveal themselves in these matrices such as the ones between a second grader classroom and a third grader classroom, and a fourth grader classroom and kindergarteners. We believe that the reasons for these interactions are some common activity between different grade classrooms and physical proximity, respectively.**The Threshold Effect**

One of the important challenges in the weighted network studies is finding the optimum threshold so the network visualizations look informative and some network measurements such as degree that are not based on weights give coherent and meaningful results. Threshold is defined as the value used in deciding to omit “insignificant” contacts from a pure network theoretic point of view rather than epidemiological. In order to find the optimum threshold we usually plot the density versus threshold graph. S2 Figure shows how the applied threshold affects the density as can be seen below and they are related to survival probability of contacts against threshold. The graph on the right is a zoomed-in version of the one on the left. One can observe from these graphs that applying a high threshold decreases the density significantly, which implies that overall structure of the network might be lost. On the other hand, applying a very low threshold is not helpful either as it does not remove insignificant, i.e., very low-duration, contacts from the network. In this study, for some network measurements and social contact network visualizations we used a threshold value of 5 minutes.

**S2 Fig. Density of the contact network versus the threshold. The panel on the right is an enlarged version for the first 20 minutes.**

Below we also show three visualizations of the social contact network of an elementary school with different threshold values of 5, 15, and 30 minutes, S3 Fig. In these visualizations we use different colors for the grades as well as stationary motes and motes worn by staff members. For a threshold of x minutes we delete all the connections in the network representing contacts with cumulative duration less than x minutes. Network visualizations show that even with a 5-minute threshold the sub-networks representing different grades are easily discernible. This is unfortunately not the case in high schools and to some extent in middle schools due to low modularity but to maintain consistency we used the same threshold value for all schools. Increasing threshold makes grade sub-networks more apparent at the expense of loosing epidemiologically meaningful mid-duration contacts.


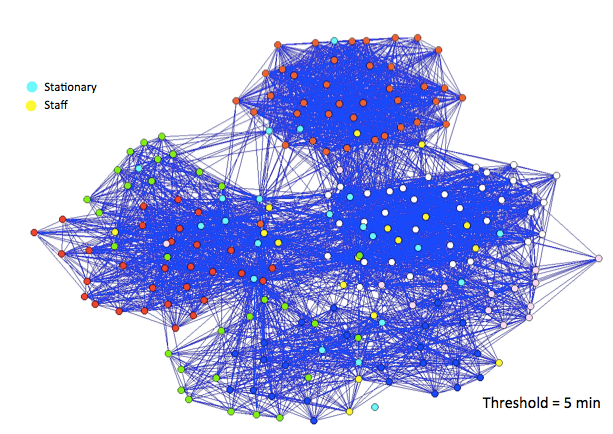

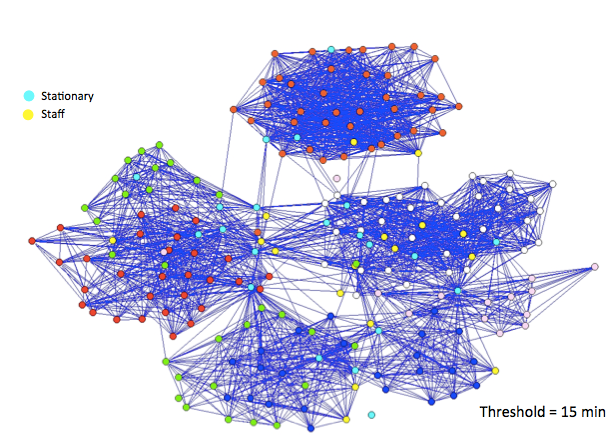


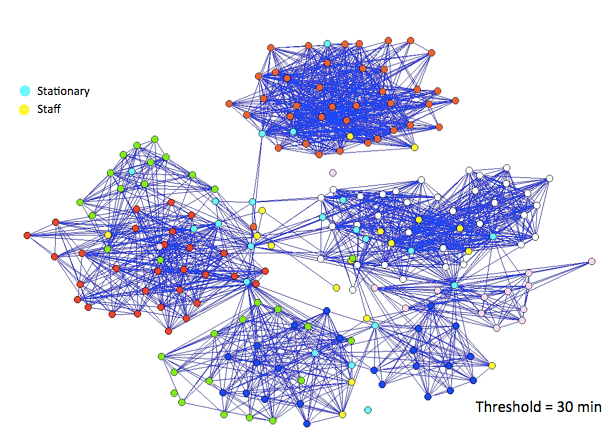


**S3 Fig. Network visualizations of a charter elementary school (C-ES) for different thresholds: 5, 15, and 30 minutes. Each node is colored according to its type: cyan (stationary motes), yellow (staff members including teachers) and other colors (grades K-4).**

**Acknowledgments**

This research was supported by Cooperative Agreement number 1U01CK00179-01 from the US Centers for Disease Control and Prevention (CDC). Its contents are solely the responsibility of the authors and do not necessarily represent the official position of CDC. We would like to thank Propel Charter Schools and Canon-McMillan School District for their participation in this research and, specifically, our coordinators for each district, Kristen Golomb and Grace Lani, respectively. Thanks also to those who helped us with mote days, Jennifer Cousins, Eva Noble, Isabel Rodriguez-Barraquer, Henrik Salje, Ben Althouse, and Andrew Azman.

**Ethics Statement**

In this study participation was voluntary and all parents were asked to fill out opt-out forms in paper form if they did not want their children to carry motes. Average opt-out ratio was 7%. The study was reviewed and approved by institutional review board (IRB) from the University of Pittsburgh (IRB# REN15020012 / PRO11120186 ), and under the US Centers for Disease Control and Prevention IRB authorization agreement. In addition, the IRBs of the two universities that collaborated on this study, the Johns Hopkins Bloomberg School of Public Health and the University of Liverpool, also reviewed and approved the study protocol. The demographics of the school populations is more urban and includes 70.5% Caucasian, 25.8% African-American, and 0.9% Asian, reflecting a more urban population, whereas Pittsburgh metro area has (population around 2.6 million): 89.8% Caucasian, 7.7% African-American, 1.1% Asian, and 0.7% Hispanic.

**Relevant Papers:**

1. Cauchemez S et al (2011). Role of social networks in shaping disease transmission during a community outbreak of 2009 H1N1 pandemic influenza. Proceedings of the National Academy of Sciences of the United States of America 108(7): 2825-2830.
2. Conlan A et al (2010). Measuring social networks in British primary schools through scientific engagement. Proc Biol Sci. 2010 Nov 3. [Epub ahead of print]
3. Eames KTD et al (2010). The impact of illness and the impact of school closure on social contact patterns. Health Technol Assess. 2010 Jul;14(34):267-312.
4. Effler PV et al (2010). Household responses to pandemic (H1N1) 2009-related school closures, Perth, Western Australia. Emerg Infect Dis. 2010 Feb;16(2):205-11.
5. Gemmetto V, Barrat A, Cattuto C. Mitigation of infectious disease at school: targeted class closure vs school closures. BMC Infectious Diseases 2014; 14:695.
6. Glass LM & Glass RJ (2008). Social contact networks for the spread of pandemic influenza in children and teenagers. BMC Public Health 8: 61.
7. Glezen WP (1996). Emerging infections: Pandemic influenza. Epidemiologic Reviews 18(1): 64-76.
8. Grassley N & Fraser C (2006). Seasonal infectious disease epidemiology. Proc Biol. Sci 273: 2541-2550.
9. Jackson C et al (2011). School closures and student contact patterns. Emerg Infect Dis. 2011 Feb;17(2):245-7.
10. Mikolajczyk et al (2008). Social contacts of school children and the transmission of respiratory- spread pathogens. Epidemiol. Infect. (2008), 136, 813–822.
11. Miller E et al (2010). Incidence of 2009 pandemic influenza A H1N1 infection in England: a cross-sectional serological study. The Lancet 375, 1100-1108.
12. Miller J et al (2010). Student behavior during a school closure caused by pandemic influenza A/H1N1. PLoS One. 2010 May 5;5(5):e10425.
13. Salathé M et al (2011). A high-resolution human contact network for infectious disease transmission. PNAS 107(51):22020-5.

1. smart.pitt.edu [↑](#footnote-ref-1)
2. <http://www.memsic.com/userfiles/files/Datasheets/WSN/telosb_datasheet.pdf> [↑](#footnote-ref-2)
3. [www.tinyos.net](http://www.tinyos.net) [↑](#footnote-ref-3)
